# Functional food mixtures: Inhibition of lipid peroxidation, HMGCoA reductase, and ACAT2 in hypercholesterolemia‐induced rats

**DOI:** 10.1002/fsn3.2051

**Published:** 2020-12-03

**Authors:** Noor Syafiqa Aqila Mohd Rosmi, Nurul Husna Shafie, Azrina Azlan, Maizaton Atmadini Abdullah

**Affiliations:** ^1^ Department of Nutrition Faculty of Medicine and Health Sciences Universiti Putra Malaysia (UPM) Serdang Malaysia; ^2^ Laboratory of UPM‐MAKNA Cancer Research Institute of Bioscience Universiti Putra Malaysia Serdang Malaysia; ^3^ Department of Pathology Faculty of Medicine and Health Sciences Universiti Putra Malaysia (UPM) Serdang Malaysia

**Keywords:** ACAT2, bioactive compounds, functional foods, HMGCR, hypercholesterolemia, lipid peroxidation

## Abstract

Mixtures of selected functional foods (MSFF) were composed of *nattokinase* (fermented soybean), red yeast rice extract, *Ginkgo biloba*, oat fiber, garlic, bee pollen, and propolis as anti‐hypercholesterolemic were studied. The goal of this study was to determine the bioactive compounds in these mixtures and their cholesterol‐lowering potential effects (biochemical profiles, lipid peroxidation, liver tissue histopathology, and enzymatic activity analysis; HMGCoA reductase and ACAT2. The LC‐MS/MS analysis showed that bioactive compounds such as Monacolin K, naringin, tocopherol, and glutamate, which have potential as anti‐hypercholesterolemic agents, were present in these functional food mixtures. MSFF supplementation at 50 mg/kg 100 mg/kg and 200 mg/kg showed substantial reductions in serum lipid profiles (TC and LDL) (*p* < .05). The serum liver profiles of AST (115.33 ± 8.69 U/L) and ALT (61.00 ± 1.00 U/L) were significantly reduced (*p* < .05) with MSFF supplementation at 200 mg/kg. MDA lipid peroxidation has also decreased significantly (*p* < .05) in serum (3.69 ± 0.42 μmol/L) and liver (15.04 ± 0.97 μmol/mg) tissues and has been shown to protect against hepatic steatosis. The significant (*p* < .05) inhibition activity of HMGCoA reductase (163.82 ± 3.50 pg/ml) and ACAT2 (348.35 ± 18.85 pg/ml) was also attributed by the supplementation of MSFF at 200 mg/kg.

## Graphical abstract

Mixtures of selected functional foods (MSFF) contained bioactive compounds (Monacolin K, naringin, tocopherol and glutamate) as anti‐hypercholesterolemic agents based on LC‐MS/MS analysis. Supplementation with MSFF were reduced the level of serum lipid profiles (TC and LDL), decreased serum liver profiles level (AST and ALT) in hypercholesterolemic rats. Lipid peroxidation was inhibited via reduction of MDA levels in serum and tissue and protect against hepatic steatosis. Greatest inhibition activities of HMGCoA reductase and ACAT2 were imposed in hypercholesterolemic rats.

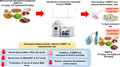

## 1 INTRODUCTION

Cardiovascular disease (CVD) is the leading cause of morbidity and mortality worldwide in most developing countries (Berzou et al., 2014). World Health Organization (2017) estimated that about 2.6 million deaths and 29.7 million disability‐adjusted life years among the global population due to diseases related to cholesterol elevation in blood. In Malaysia, the prevalence of diagnosed with hypercholesterolemia among adults was 40.3% according to National Health and Morbidity Survey (National Health & Morbidity Survey, 2019).

Hypercholesterolemia is a condition that refers to a metabolic disorder that may result in elevated concentration of plasma low‐density lipoprotein (LDL) cholesterol (Adekiya et al., 2018; Mu et al., 2017). Hypercholesterolemia has become a primary risk factor for the pathogenesis of cardiovascular diseases such as the development of atherosclerosis, hyperlipidemia, coronary heart diseases, ischemic heart diseases, and stroke due to the presence of high levels of cholesterol in the blood (Cheong et al., 2018).

Nowadays, conventional synthetic lipid‐lowering drugs such as fibrates, statins, and bile acid sequestrants had been acknowledged for the treatment of hypercholesterolemia. However, these medications still have limited efficacy and severe side effects including myopathy, rhabdomyolysis, and polyneuropathy (Moosmann & Behl, 2004). It is important to find an alternative for the treatment of hypercholesterolemia from natural sources due to their potential as anti‐hypercholesterolemic agents compared to synthetic drugs that have side effects for long‐term consumption.

Natural ingredients such as *nattokinase* (fermented soybean product), red yeast rice extract, *Ginkgo biloba*, oat fiber, garlic, bee pollen, and propolis have potential effects as anti‐hypercholesteraemic agents. The bioactive compounds such as Monacolin K, a product from fermented red yeast rice resembled similar properties as lovastatin potentially decrease the level of HMGCoA reductase activity (Ajdari et al., 2014). Besides, naringin from bee pollen and propolis as antioxidant ability to scavenge against free radicals and block the activity of HMGCoA reductase (Sobral et al., 2017). Tocopherol derivatives, tocotrienols also provide hepatoprotective properties against fatty liver diseases and interact with plant sterols as soybeans and oat fiber to retard the synthesis of cholesterol (Guo et al., 2014; Hoene et al., 2018). Glutamate derivatives, glutamic acid that mainly found in soybeans and oat help to down‐regulate the cholesterol biosynthesis by suppressing LDL level in rats (Wan Saidatul Syida et al., 2018).

Previous studies also reported the anti‐hypercholesterolemic properties of the single ingredients as *nattokinase* (fermented soybean*)* is regarded as an anti‐atherosclerotic agent to suppress intimal thickening in rats (Chen et al., 2018). Besides, red yeast rice extract can decrease blood cholesterol by reducing lipid peroxidation (Yeap et al., 2014). *Ginkgo biloba* may stimulate the production HDL level (Kang, 2017). Oat fiber aid in lowering total cholesterol levels due to its role in altering the metabolism of bile acids. Garlic has *allicin* compound that may reduce streaks formation (atherosclerosis) and bee pollen control the elevation of lipid and cholesterol at the normal level and prevent clumping of blood platelets (Komosinska‐Vassev et al., 2015). Propolis can suppress the level of triglyceride in the rats (Albokhadaim, 2015).

Previous studies have shown promising effects of each constituents of these functional foods in alleviating hypercholesterolemia in vivo (Bharti et al., 2017; Desamero et al., 2017; Weng et al., 2017; Xu et al., 2014; Yang et al., 2018). Predicated upon that the present study aimed to determine synergistic effects of bioactive compounds in mixtures of these selected functional foods in hypercholesterolemia‐induced rats.

## 2 MATERIALS & METHOD

### 2.1 Chemicals and reagents

ELISA Kits (HMGCoA reductase and ACAT2) (Sunlong Biotech Co. Ltd), thiobarbituric acid (TBA) (Sigma‐Aldrich), trichloroacetic acid (TCA) (Sigma‐Aldrich), 1, 1, 3–3‐tetraethoxypropane (TEP) (Sigma‐Aldrich), 1% high cholesterol diet (Envigo Teklad), normal rat diet (Takrif Bestari Enterprise), Simvastatin (10 mg/kg)(Pharmaniaga Manufacturing Berhad).

### 2.2 Equipment and instruments

Chemistry auto‐analyzer machine (Roche Diagnostic), cholesterol strips (Accutrend Plus System, Roche Diagnostic), Cobas meter (Roche Diagnostic), centrifuge machine (X‐22R, Beckman Coulter), LC‐MS/MS mass spectrometer coupled to HPLC (Ultimate 3,000 series, Thermo Scientific Dionex) with C18 column (Thermo Scientific Hypersil Gold).

### 2.3 Functional foods sample

The investigational functional foods product contained mixtures of different types of raw extracts; such as bee pollen (88.2 mg), propolis (84.0 mg), *nattokinase* (84.0 mg), oat fiber (42.0 mg), water extract of *Ginkgo biloba* (25.2 mg), thin layer chromatography (TLC) extract of red yeast rice (33.6 mg), and ethanol extract of garlic bulb powder (63.0 mg).

### 2.4 Composition of 1% HCD diet

The diet was based on a purified atherogenic diet that consisted of 1% (10 g/kg) of cholesterol, 0.5% (5 g/kg) of sodium cholate.

Fatty acid distribution (62% of saturated fat; 31% of monounsaturated fat; 7% of polyunsaturated fat). Nutrition composition of 1% high cholesterol diet (carbohydrate [49.1%]: sucrose [450 g/kg]; cellulose [52.22 g/kg]; corn starch [50 g/kg]), (fat [34.2%]; anyhydrous milk fat [150 g/kg]; corn oil [10 g/kg]), and protein (16.6%).

### 2.5 Identification of bioactive compounds by LC‐MS/MS

Briefly, 10 mg extract from mixtures of selected functional foods (MSFF) were macerated with 1 ml of aqueous solutions of methanol and filtered using a 0.2 mm chromatographic filter and LC‐MS/MS operating system that coupled to HPLC (Matei, Gatea, & Radu, 2015). The chromatographic separations were accomplished by C18 column with particle size at 1.9 µm, (length: 100 mm × diameter: 2.1 mm) and the column temperature was kept at 40°C. Solution A (Water + 0.1% Formic Acid) and Solution B (Acetonitrile) were served as mobile phases at flow rate of 0.4 ml/min. The injection volume was 2.0 µl and the column temperature was set at 40°C. The mass spectrometer was coupled with heated electrospray ionization (HESI) sources that run in negative and positive ion modes with high sensitivity. Data were analyzed and searched by comparing parent ion fragmentation pattern m/z with database (PubChem, Mass Bank, and Chem Spider).

### 2.6 Experimental animals

Thirty‐six Sprague Dawley rats (150–250 g) had been acclimatized for a week by given normal chow and tap water ad libitum. Rats were housed in individual stainless cages (3 rats/cage), fully ventilated and maintained at room temperature with 12 hr light‐dark cycle at Animal House, Faculty of Medicine and Health Sciences, Universiti Putra Malaysia (UPM). Animal ethics had been approved by the Institutional Animal Care and Use Committee (IACUC), Universiti Putra Malaysia (UPM) (AUP No: UPM/IACUC/AUPRO84/2018). The rats were randomly divided into six groups of six rats each (*n* = 6). The rats were divided into two groups (normal control [NC] and high cholesterol fed rats [HCD]) for initial 4 weeks. After 4 weeks on high cholesterol diet, the group were divided into five groups: 1% HCD, 1% HCD + Simvastatin (10 mg/kg), 1% HCD + MSFF (50 mg/kg), 1% HCD + MSFF (100 mg/kg) and 1% HCD + MSFF (200 mg/kg). At the end of the experimental period, rats were anesthetized with an intraperitoneal injection of ketamine (100 mg/kg) and xylazine (10 mg/kg), serum and tissues were collected for further analysis.

### 2.7 Measurement of body weight changes and absolute liver weight

The measurement of the body weight of all groups was taken regularly at week 0, week 4, and week 8. The absolute liver weight of all groups was measured at end of week 8 based on the formula according to Adeneye et al., (2010).

### 2.8 Biochemical analysis

The biochemical analysis of serum lipid profiles (TC, TG, LDL, and HDL), serum kidney profiles (creatinine, urea, and uric acid), and serum liver profiles (ALT and AST) were determined using Chemistry Auto‐analyzer machine (Roche Diagnostic) (Puttaswamy & Urooj, 2016; Yeap et al., 2015).

### 2.9 Determination of serum and tissue MDA levels

Briefly, 125 μl of the supernatant of the liver was mixed with 175 μl of 20% trichloroacetic acid (TCA) containing 1% butylhydroxytoluene (BHT) and centrifuged (1,000 ×*g*, 10 min, 4°C). Then, 200 μl of supernatant was mixed with 40 μl of HCl (0.6 M), 160 μl of thiobarbituric acid (TBA) (0.72 mM), and mixtures were heated at 80°C for 10 min and measured at 530 nm. Tetramethoxypropane was used as a standard. TBARS results were expressed as micromole MDA production per liter (µmol/L) serum and (µmol/mg) tissue (Samout et al., 2015).

### 2.10 Histopathological analysis of liver tissues

Liver tissues were fixed in 10% formalin phosphate buffer solution, dehydrated, paraffin‐embedded, and archived. All samples had been undergone sectioning process and mounted on aminopropyltriethoxysilane‐coated slides. Next, deparaffinization in xylene, sections rehydrated, stained with hematoxylin and eosin (H & E), and examined by light microscopy (Yeh et al., 2010).

### 2.11 Enzymatic assays (HMGCoA reductase and ACAT2)

Enzymatic assays of HMGCoA reductase and ACAT2 in rat's liver were determined respectively using ELISA Kits according to the manufacturer's instruction and measured at 450 nm.

#### 2.11.1 Data analysis

All results in this experiment were expressed as mean ± standard error (*SEM*). One‐way ANOVA (Tukey test) was used for all experiments. Two‐way repeated‐measures ANOVA by post hoc Bonferroni test was analyzed for body weight changes and all values with *p* < .05 were considered significant.

## 3 RESULTS

### 3.1 Identification of bioactive compounds

**FIGURE 1:**
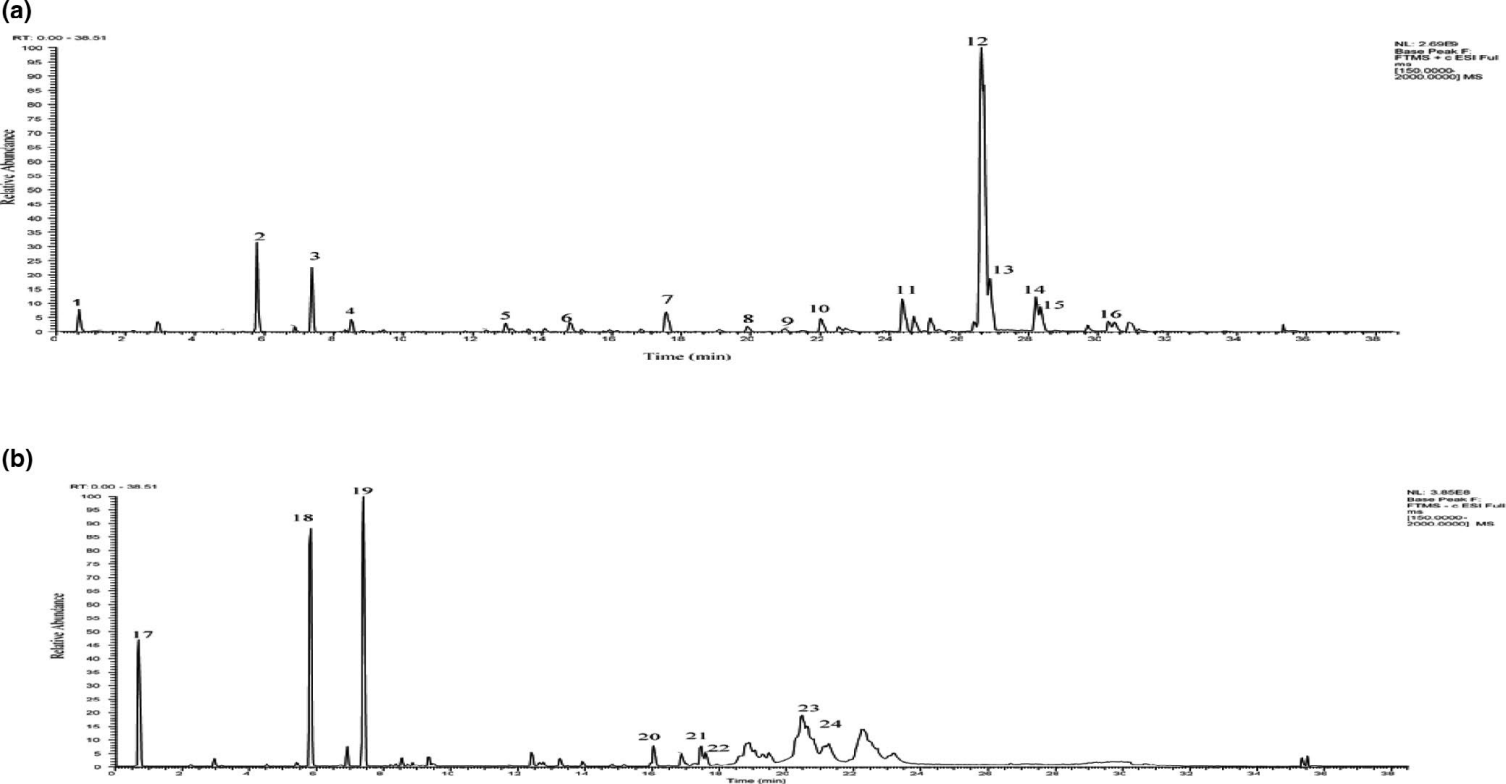
(a) Liquid chromatography–mass spectrometry chromatographic profiles of MSFF + positive ion mode. (b) Liquid chromatography–mass spectrometry chromatographic profiles of MSFF – negative ion mode

Results showed peaks formed on chromatogram were appeared at positive (Figure 1a) and negative ion (Figure 1b) modes were detected for LC‐MS/MS analysis depending on different chemical properties of MSFF active compounds accordingly. Table 1 listed the peak, retention time, compounds, integrated peak area, ion mode, and precursor ion for m/z value, molecular weight, and chemical formula respectively. About twenty‐four (24) of major constituents were identified or tentatively characterized in MSFF combinations. They included monosaccharides (simple sugar derivatives) as hexitol (Compound 1) and Bis (4‐ethylbenzylidene) sorbitol (Compound 6). Furthermore, alkaloids groups included methyl‐1‐pentyl‐1H‐indole‐3‐carboxylate (Compound 2) and 4‐(2‐Aminoethylcarbamoylamino)‐N‐ piperidine‐1‐carboxamide (Compound 18). Besides, there was also the availability of active secondary metabolites consisted of monacolin K (Compound 7), guaiazulene (Compound 8), tocopherol (Compound 15), naringin (Compound 17).

**TABLE 1:** Bioactive compounds identification based on LC‐MS/MS

Peak	Retention time (R_t_) (min)	Compounds	Integrated Peak area	Ion mode	Precursor ions (m/z)	Molecular weight (MW) (g/mol)	Formula
1	0.66	Hexitol	1,239,991	[M + H]^+^	183.09	182.0790	C_6_H_14_O_6_
2	5.78	Methyl−1‐pentyl−1H‐indole−3‐carboxylate	3,527,172	[M + 2H]^+2^	246.149	490.284	C_15_ H_19_ NO_2_
3	7.36	1,8,15,22‐Tetraazacyclooctacosane−2,9,16,23‐tetrone	3,425,388	[M + H]^+^	453.34	452.337	C_24_ H_44_ N_4_O_4_
4	8.50	Dibutyl *n*‐acetylvalylglutamate	708,239	[M + Na]^+^	401.27	400.510	C_20_H_36_N_2_O_6_
5	12.95	Dodecyldiethanolamine	653,766	[M + H]^+^	274.27	273.26	C_16_ H_35_ NO_2_
6	14.79	Bis(4‐ethylbenzylidene) sorbitol	773,023	[M + Na]^+^	415.2037	414.2056	C_24_ H_30_ O_6_
7	17.56	Monacolin K	1,458,448	[M + Na]^+^	405.2647	404.25	C_24_H_36_O_5_
8	19.91	Guaiazulene	118,914	[M + H]^+^	199.148	198.14145	C_15_ H_18_
9	20.98	4‐Hexadecylphenol	67,220	[M + NH4]^+^	319.300	318.2938	C_22_ H_38_ O
10	22.02	Hexadecanamide	931,796	[M + H]^+^	256.2635	255.44	C_16_ H_33_ NO
11	24.37	Stearamide	3,385,704	[M + H]^+^	284.296	283.288	C_18_H_37_NO
12	26.65	(4‐hexyldecyl) benzene	34,514,333	[M + H]^+^	303.306	302.306	C_22_H_38_
13	26.88	(‐)‐Cholesteryl acetate	433,417	[M + H]^+^	429.3727	428.3654	C_29_ H_48_ O_2_
14	28.20	Docosanamide	3,535,473	[M + H]^+^	340.3574	339.3501	C_22_H_45_NO
15	28.35	Tocopherol	145,079	[M + H]^+^	431.38	430.38	C_29_ H_50_ O_2_
16	30.29	Lignoceric acid	19,120.5	[M + H]^+^	368.60	369.37	C_24_ H_48_ O_2_
17	0.68	Naringin	8,665,299	[M‐H]^‐^	579.175	580.179	C27H32O14
18	5.79	4‐(2‐Aminoethylcarbamoylamino)‐*N*‐ piperidine−1‐carboxamide	15,334,854	[M‐H]^‐^	514.32	515.33	C25 H41 N9 O3
19	7.37	Unidentified	18,087,179	[M‐H]^‐^	385.234	741.4987	C_44_ H_71_ NO_6_ S
20	16.02	N‐Cyclododecyl−1,9‐dimethyl−4‐oxo−1,4‐dihydropyridopyrimidine−2‐carboxamide	175,221	[M‐H]^‐^	421.259	422.266	C_25_ H_34_ N_4_ O_2_
21	17.43	Dodecyl sulfate	126,321	[M‐H]^‐^	265.147	266.152	C_12_ H_26_ O_4_ S
22	18.85	4‐Undecylbenzenesulfonic acid	8,761,334	[M‐H]^‐^	311.223	312.1759	C_17_ H_28_ O_3_ S
23	20.44	4‐Dodecylbenzenesulfonic acid	1,874,807	[M‐H]^‐^	325.14	326.191	C_18_ H_30_ O_3_ S
24	20.57	L‐Palmitoylcarnitine	54,791	[M‐H]^‐^	398.00	399.33	C_23_H_45_NO_4_

Furthermore, fatty acids profiles such as hexadecanamide (Compound 10), stearamide (Compound 11), docosanamide (Compound 14), lignoceric acid (Compound 16), L‐palmitoylcarnitine (Compound 24). Another compound also represented from phenol group which was known as 4‐hexadecylphenol (Compound 9) and phenyl group, (4‐hexyldecyl) benzene (Compound 12). MSFF also exhibited the presence of sterol as cholesteryl acetate (Compound 9), amino acid group as dibutyl n‐acetylvalylglutamate (Compound 4), and nucleotides as N‐cyclododecyl‐1, 9‐dimethyl‐4‐oxo‐1,4‐dihydropyridopyrimidine‐2‐carboxamide(Compound 20). Organosulfur compounds had a presence with the availability of 4‐undecylbenzenesulfonic acid (Compound 22) and 4‐dodecylbenzenesulfonic acid (Compound 23).

### 3.2 Measurement of body weight changes and absolute liver weight

**FIGURE 2:**
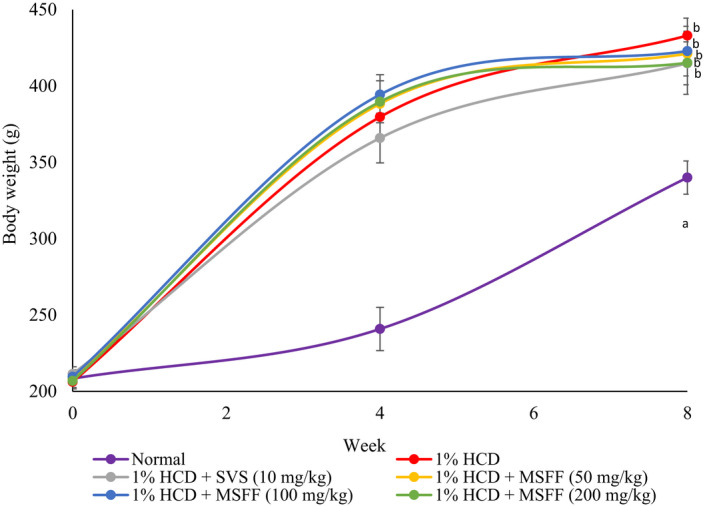
Body weight changes during week 0 (initial weight), week 4 (after induction) and week 8 (after treatment) for all experimental rats. Values are expressed as mean ± standard error, *SEM* (*n* = 6). Significance was measured by performing one‐way ANOVA followed by Bonferroni's post hoc test, *p*‐value for ANOVA for repeated measures was given (*p* < .05)

All groups showed almost similar starting point of initial body weight at week 0 respectively (Figure 2). After 4 weeks of induction with 1% high cholesterol diet (HCD), all induction groups showed a significant (*p* < .05) increased of body weight compared to normal group (Figure 2). There were no significant differences in body weight between all 1% HCD induction groups and control group (Figure 2). Week 8 (duration after treatment) revealed that 1% HCD group was showed a significant (*p* < .05) increased (433.00 ± 11.41 g) of body weight than normal group (340.00 ± 10.87 g). There were no significant different (*p* > .05) of body weight changes of all treated rats with MSFF at 50 mg/kg (421.16 ± 16.51 g) 100 mg/kg (422.83 ± 16.26 g) , 200 mg/kg (415.17 ± 20.63 g) and treatment with SVS at 10 mg/kg (414.83 ± 14.04 g) compared to 1% HCD group (433.00 ± 11.41 g).

**FIGURE 3:**
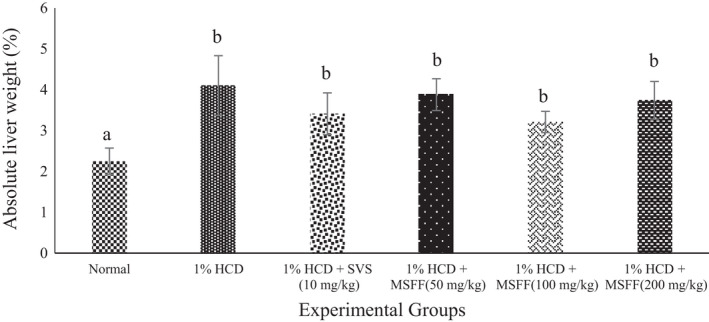
Effects of different treatments given on absolute liver weight (%) in all experimental rats. Values are expressed as mean ± standard error, *SEM* (*n* = 6). Means values with different letters were significantly different at level (*p* < .05) between all groups

Besides, 1% HCD group and all treated groups were showed a significant (*p* < .05) increased of absolute liver weight than normal group after week 8 (Figure 3). There were no significant different (*p* > .05) of absolute liver weight of all treated rats with MSFF at 50 mg/kg (3.88 ± 0.39%), 100 mg/kg (3.21 ± 0.26%), 200 mg/kg (3.74 ± 0.46%) and treated rats with SVS at 10 mg/kg (3.41 ± 0.51%) when compared to 1% HCD group (4.10 ± 0.73%).

### 3.3 Biochemical analysis of serum lipid profiles (TC, TG, LDL, and HDL)

**FIGURE 4:**
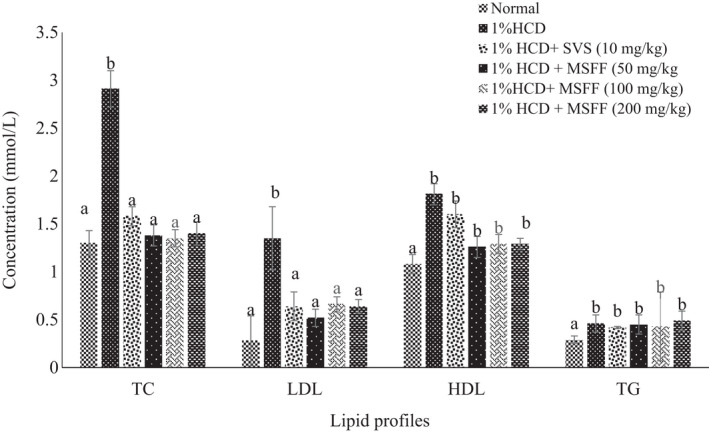
Effects of different treatments given on serum lipid profiles (TC, LDL, HDL, TG) in all experimental rats. Values are expressed as mean ± standard error, *SEM* (*n* = 6). Means values with different letters were significantly different at level (*p* < .05) between all groups

As shown in Figure 4 1% HCD group exhibited a significant (*p* < .05) increased of TC level (2.91 ± 0.19 mmol/L) and LDL (1.35 ± 0.33 mmol/L) than normal group. Treatment of MSFF at different doses (50 mg/kg, 100 mg/kg, and 200 mg/kg) was found a significant (*p* < .05) decreased of TC and LDL levels compared to 1% HCD group. Treated rats with SVS at 10 mg/kg showed no significant difference (*p* > .05) of TC (1.58 ± 0.10 mmol/L) and LDL (0.64 ± 0.15 mmol/L) when compared to all treated rats with MSFF. However, the treatment of MSFF at different doses showed no significant difference in TG and HDL levels when compared with 1% HCD group (Figure 4).

### 3.4 Biochemical analysis of serum kidney and liver profiles

**FIGURE 5:**
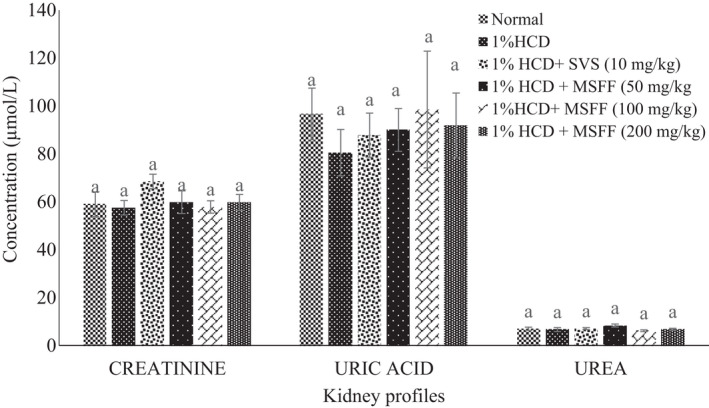
Effects of different treatments given on serum kidney profiles (creatinine, uric Acid, urea) in all experimental rats. Values are expressed as mean ± standard error, *SEM* (*n* = 6). Means values with different letters were significantly different at level (*p* < .05) between all groups

**FIGURE 6:**
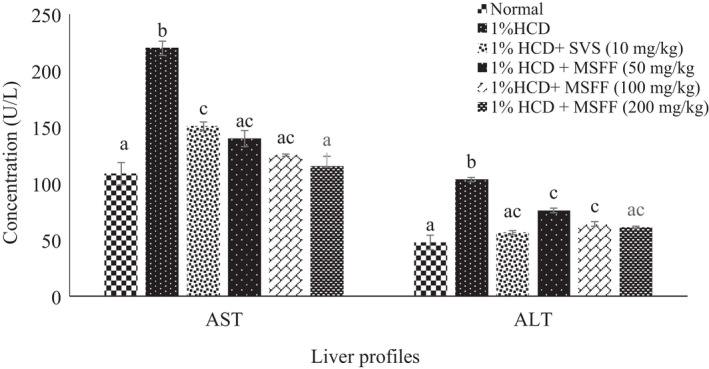
Effects of different treatments given on serum liver profiles (AST, ALT) in all experimental rats. Values are expressed as mean ± standard error, *SEM* (*n* = 6). Means values with different letters were significantly different at level (*p* < .05) between all groups

Figure 5 shows there was no significant effect (*p* > .05) between 1% HCD group and all treated rats with SVS (10 mg/kg) and MSFF (50 mg/kg, 100 mg/kg and 200 mg/kg) on serum kidney profiles (creatinine, urea, and uric acid). For serum liver profiles, serum AST (219.67 ± 6.36 U/L) and serum ALT (103.33 ± 1.86 U/L) were showed a significant (*p* < .05) increased in 1% HCD group than normal group (Figure 6). Treated rats with MSFF at 200 mg/kg was showed a significant (*p* < .05) decreased of AST (115.33 ± 8.69 U/L) and ALT (61.00 ± 1.00 U/L) values than 1% HCD group. However, treated rats with SVS at 10 mg/kg showed a significant (*p* < .05) increased of AST value (150.67 ± 3.76 U/L) compared to treated rats with MSFF particularly at 200 mg/kg (115.33 ± 8.69 U/L). Besides, we have also demonstrated that the MSFF was less toxic when compared to SVS using the zebrafish toxicity assay (unpublished data).

### 3.5 Serum and tissue malondialdehyde (MDA) levels

**FIGURE 7:**
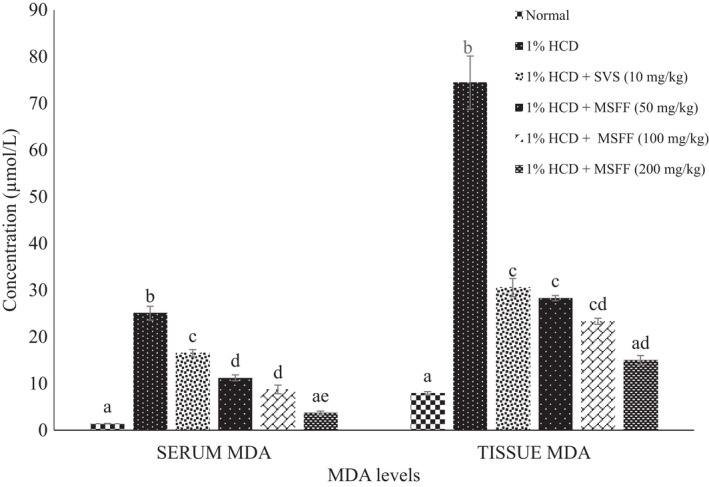
Effects of different treatments given on serum and tissue malondialdehyde (MDA) in all experimental rats. Values are expressed as mean ± standard error, *SEM* (*n* = 6). Means values with different letters were significantly different at level (*p* < .05) between all groups

Serum MDA level was found a significant increased (*p* < .05) in 1% HCD group (25.06 ± 1.49 µmol/L) compared to normal group (1.41 ± 0.05 µmol/L) (Figure 7). All treated rats with MSFF at 50 mg/kg (11.22 ± 0.64 µmol/L), 100 mg/kg (8.74 ± 0.92 µmol/L) and 200 mg/kg (3.69 ± 0.42 µmol/L) and also SVS (16.50 ± 0.77 µmol/L) were exhibited a significant decreased (*p* < .05) in serum MDA levels compared to 1% HCD group (25.06 ± 1.49 µmol/L).

For tissue MDA level, 1% HCD group had a significant (*p* < .05) increased (74.42 ± 5.72 µmol/mg) compared to normal group (7.97 ± 0.30 µmol/mg) (Figure 7).Treated rats with all doses of MSFF and SVS showed significant (*p* < .05) decreased of MDA levels compared to 1% HCD group. Furthermore, there was a significant (*p* < .05) decreased of tissue MDA levels in treated rats with MSFF at highest dose (200 mg/kg; 15.04 ± 0.97 µmol/mg) when compared with SVS (30.55 ± 1.95 µmol/mg).

### 3.6 Histopathological analysis of liver tissues

**FIGURE 8:**
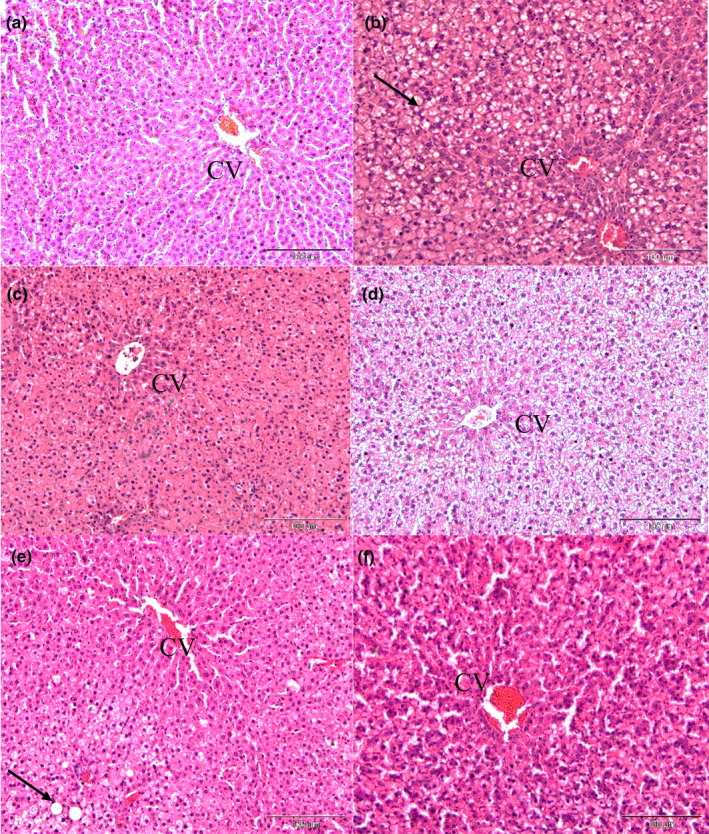
Effect of different treatments on liver tissues histological analysis of all experimental rats (*n* = 6). Liver sections were stained with hematoxylin and eosin (H&E) and examine under light microscope (100X). (a) Normal hepatic strands around central vein (CV). (b) 1% HCD group showing visible fat vacuoles, microvascular and macrovascular hepatic steatosis with ballooned hepatocytes. (c) 1% HCD + SVS (10 mg/kg) showing ballooned hepatocytes without visible hepatic steatosis. (d) 1% HCD + MSFF (50 mg/kg) has aligned strands of hepatocytes but enlarge fat vacuoles. (e) 1% HCD + MSFF (100 mg/kg) has a few visible macrovascular hepatic steatosis. (f) 1% HCD + MSFF (200 mg/kg) has no visible hepatic steatosis and hepatocytes strands re‐aligned as normal

The hepatic strands of hepatocytes indicated the normal alignment in absence of ballooned fat vacuoles surrounded central vein (CV) in the liver tissue of normal rats (Figure 8a) (Brunt et al., 1999; Takashi & Fukusato, 2014). Normal group had been scored as 0 due to absence of hepatic steatosis indicated as healthy (<5%) (Table 2). Besides, there were the ubiquitous and visible of numerous microvascular and macrovascular hepatic steatosis and ballooning of liver hepatocytes around central vein followed by disruption of by histoarchitectural distortion on hepatic strands and congested central vein in 1% HCD group (Figure 8b). This group was scored as 3.00 ± 0.00 indicating the severity (>66%) and the significant highest score (*p* < .05) compared to normal group.

**TABLE 2:** Hepatic steatosis scoring for all experimental rats

Group/Week	Hepatic steatosis score
Normal	0.00 ± 0.00^a^
1% HCD	3.00 ± 0.00^b^
1% HCD + SVS (10 mg/kg)	0.67 ± 0.33^ad^
1% HCD + MSFF (50 mg/kg)	2.00 ± 0.00^c^
1% HCD + MSFF (100 mg/kg)	1.00 ± 0.00^d^
1% HCD + MSFF (200 mg/kg)	0.33 ± 0.33^ad^

Note

Values are expressed as mean ± standard error, *SEM* (*n* = 6). Means values with different letters were significantly different at level (*p* < .05) between all groups. 1% HCD, 1% high cholesterol diet, MSFF, mixtures from selected functional foods, SVS, simvastatin. Scoring of hepatic steatosis based on percent of hepatocytes in the biopsy involved (<5%: 0 (healthy), 5%–33%:1 (mild) , 34%–66%:2 (moderate) and >66%: 3 (severe) (Brunt et al., 1999).

The enlargement of fat vacuoles with clear ballooned hepatocytes was observed and the development of microvascular hepatic steatosis in rats after treatment with MSFF (50 mg/kg) (Figure 8d. It was scored as 2.00 ± 0.00 as moderate classification that falls at this range (34%–66%) and significantly (*p* < .05) highest compared to other MSFF doses (100 mg/kg and 200 mg/kg). However, the hepatic strands surrounded and nearest the central vein (CV) were started to re‐align into normal structures respectively at MSFF (100 mg/kg) (Figure 8e).

This group was scored at 1.00 ± 0.00, which classified as mild, (5%–33%). Fat droplets were reduced and ballooned hepatocytes had returned into normal strands although a slight irregular form of sinusoids after treatment with MSFF 200 mg/kg (Figure 8f). Treatment with SVS at 10 mg/kg showed no development of hepatic steatosis on the surface of ballooned hepatocytes (Figure 8c). Both of these groups had been scored as 0 to represent significantly (*p* < .05) effective to mediate hepatic steatosis compared to 1% HCD group.

### 3.7 Enzymatic activities of HMGCoA reductase and ACAT2

**FIGURE 9:**
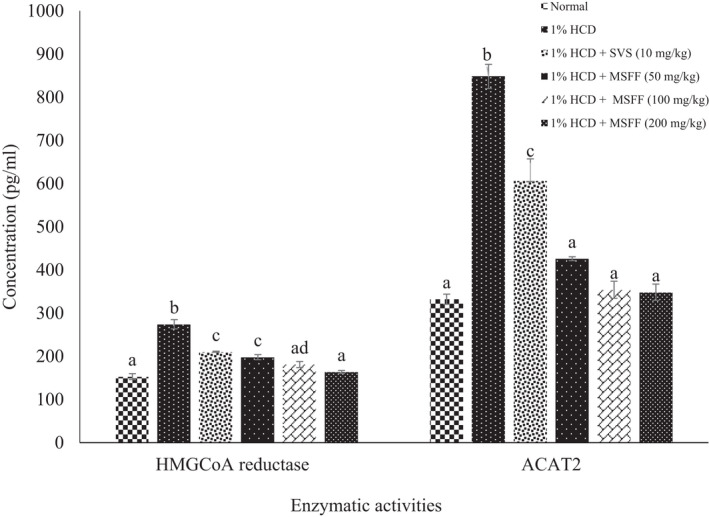
Effects of different treatments given on enzymatic activities of (HMGCoA reductase and ACAT2) in all experimental rats. Values are expressed as mean ± standard error, *SEM* (*n* = 6). Means values with different letters were significantly different at level (*p* < .05) between all groups

HMGCoA reductase activity was significant (*p* < .05) increased in 1% HCD group (274.24 ± 10.86 pg/ml) compared to normal group (152.98 ± 6.84 pg/ml). Treated rats with MSFF at 50 mg/kg (198.13 ± 5.86 pg/ml); 100 mg/kg (180.98 ± 6.97 pg/ml) and 200 mg/kg (163.82 ± 3.50) were showed a significant (*p* < .05) decreased compared to 1% HCD group (Figure 9). The activity of HMGCoA reductase was found a significant (*p* < .05) decreased in MSFF treated rats at 100 mg/kg and 200 mg/kg when compared to SVS groups but no significant different (*p* > .05) when compared to normal group.

As shown in Figure 9, ACAT2 activities showed a significant (*p* < .05) increased in 1% HCD group (848.17 ± 28.15 pg/ml) compared to normal group (332.18 ± 11.86 pg/ml). Treated rats with MSFF at doses of 50 mg/kg (425.93 ± 4.63 pg/ml), 100 mg/kg (354.23 ± 19.68 pg/ml) and 200 mg/kg (348.350 ± 18.85 pg/ml) had demonstrated their potential to exhibit a significant (*p* < .05) inhibition of ACAT2 activities compared to treated rats with simvastatin at 10 mg/kg (605.82 ± 51.47 pg/ml) and 1% HCD group (848.17 ± 28.15 pg/ml).

## 4 DISCUSSION

Combination of functional foods becomes favorable and accessible treatment against hypercholesterolemia. The functional ingredients consisted of bioactive components to provide health‐promoting action in the management of cardiovascular diseases. In this study, the identified bioactive compounds in MSFF through LC‐MS/MS application such as the presence of Monacolin K, guaiazulene, tocopherol, naringin, and glutamate were highlighted due to their potential to exhibit protective mechanism through anti‐oxidative and anti‐inflammatory properties in mediating hypercholesterolemia. It suggested the availability of bioactive compounds from MSFF might be influenced by the origin of each functional foods, growth time, storage condition, extraction method, decoction method, and product batch (Lu et al., 2013).

Feeding with 1% of high cholesterol diet had induced hypercholesterolemia with contributed to body and liver weight gain, impaired serum lipid profiles, the elevation of liver enzymatic activities, and oxidative stress. Composition of this diet that rich in 62% saturated fatty acid may cause greater body fat deposition in rats (Kai et al., 2015). Besides, 1% cholesterol would disrupt the normal physiological function of the liver to metabolize lipid and triggered the accumulation of fatty acid on hepatocytes (Cheong et al., 2018). Therefore, these conditions eventually elevated total cholesterol production and LDL in circulating blood.

Administration of MSFF at 50 mg/kg, 100 mg/kg, and 200 mg/kg were found to maintain body weight gain due to the availability of tocopherol and lignoceric acid compound. Tocopherol would possess hepatoprotective effect against fatty liver disease based on previous studies (Górnicka et al., 2019). Besides, lignoceric acid was classified as very long saturated fatty acids (VLSFA) found to be beneficial in improving metabolic disorders and cardiovascular diseases through weight gain control (Lee et al., 2015). Simvastatin (SVS) treatment at 10 mg/kg also involved in weight control but the mechanism of weight reduction was till unclearly defined because it mainly involved in the structural damage in muscles (Suzuki et al., 2018).

High cholesterol feeding at 1% becomes as an extrinsic inducer to catalyst elevation of serum TC, LDL, and TG. The additional bile salts derived from (0.5%) of sodium cholates which act as primary bile acids and deoxycholic acid as secondary bile acid thus, increasing the capacity of the liver to down‐regulate bile acid synthesis that facilitates the excretion of cholesterol (Hofmann & Hagey, 2008). Besides, the increment of cholesterol levels in the blood may trigger inflammation at kidneys and stimulate renal oxidative stress (Rajeswari et al., 2017). The deposition of fatty acid on the liver had triggered the elevation of serum AST and ALT in hypercholesterolemic conditions to indicate as liver injury (Adekiya et al., 2018).

Besides, the production of cholesterol biosynthesis could be suppressed by inhibiting the enzymatic activities of HMGCoA reductase activity. Inhibition of ACAT2 enzymatic activities also may prevent the esterification of free cholesterol before being transported in form of VLDL into LDL to be delivered through all parts of the body system (Nguyen et al., 2019). Oxidative stress had triggered the production of MDA levels in serum and tissue in hypercholesterolemic rats that might facilitate the development of hepatic steatosis and toxic to cells (Repetto et al., 2012).

Cholesterol‐lowering effects of bioactive compounds produced by combination of MSFF may exert curative action on hypercholesterolemia at different doses (50 mg/kg, 100 mg/kg, and 200 mg/kg). Monacolin K was known as a secondary metabolite from a fraction of red yeast rice extract had possessed lipid‐lowering properties via inhibition of HMGCoA reductase activities to retard cholesterol production (Ajdari et al., 2014). Besides, naringin majorly found in propolis and bee pollen exhibited strong antioxidant properties that belong to the flavonoid compound would share a similar mechanism as Monacolin K with availability of tocopherol in reducing cholesterol level (Pengnet et al., 2019; Sobral et al., 2017). In our study, combination of MSFF at lower doses (50 mg/kg) had caused a greater significant (*p* < .05) reduction of TC level (52.57%) and LDL (61.48%) compared to previous study that reported the single administration of red yeast rice extract at 60 mg/kg had decreased TC (21.80%) and LDL level (60.81%) in treated rats (Yeap et al., 2014).

Naringin, glutamine, and tocopherol compound in MSFF particularly at 100 mg/kg and 200 mg/kg also were regarded as hepatoprotective because it might scavenge free radicals to protect the liver cells by regulating the optimum serum liver profiles of ALT and AST up to more than 40% against oxidative stress and prevent the development of hepatic steatosis based on our histopathological evaluation (Adil et al., 2016; Sharma et al., 2011). However, another study found that single administration of propolis and tocopherol derivatives at 200 mg/kg only managed to reduce values of ALT (36.3%) (22.30%) and AST (29.0%) (21.47%) which elucidated a smaller changes than using MSFF treatment in hypercholesterolemic rats (Hassan Abd et al., 2017; Jayusman et al., 2017).

Furthermore, combination of MSFF had imposed stronger antioxidant capacity to protect the cell membrane through glutathione peroxidase pathway against oxidation together with naringin, glutamate, and guaiazulene by inhibiting more than 80% serum and tissue MDA production in hypercholesterolemic rats (Pavadhgul et al., 2019). The percentage of reduction using MSFF treatment at 200 mg/kg was reached almost doubled value as compared to single administration of oat extract that was found to decrease serum (45.24%) and tissue (37.89%) MDA level in rats at highest doses in recent studies (AlMalki, 2013; Aly, 2012). Previous studies also found that single propolis treatment at 100 mg/kg and 200 mg/kg only caused a significant (*p* < .05) reduction of liver tissue MDA by 4.83% and 11.8% in treated rats when compared at similar doses (Kaya et al., 2019; Selamoglu et al., 2015).

The availability of Monacolin K, naringin, and glutamate in combination of functional foods were directly contributed to inhibit the activities of HMGCoA reductase respectively into 40.26% for dose up to 200 mg/kg. In addition, *nattokinase* from soybean that consisted glutamate can accelerate production of GABA and isoflavones aglycone that suppress protein mass of HMGCoA reductase that would facilitate this mechanism of action (Liu et al., 2019).This could be revealed that previous study reported that up to 200 mg/kg administration of single fermented soybean extract was found to inhibit HMGCoA reductase activity by 28.30% in treated rats which represented only at almost half percentage of reduction compared to our data (Pyo & Seong, 2009).

Naringin has its part of aglycone, naringenin might interact together with tocopherol, isoflavones and phytosterols content from soybean fraction in MSFF to exert greater inhibition of ACAT2 activities range from 49.7% until 59.0%. Previous study reported that ACAT2 activities were inhibited at smaller range for treatment using single naringenin (35.3%), and oat extract (17.0%) at 100 mg/kg dose (ElRabey, 2013; Wilcox et al., 1999). It revealed that combination of these compounds had powerful ability to suppress the expression of microsomal triglyceride transfer protein (MTP) that involved in packaging cholesterol ester (CE) into chylomicrons from ACAT2 activity (Borradaile et al., 2002; Zeka et al., 2017). Hence, this process directly lowers the total cholesterol level and cholesterol absorption in the regulation of hypercholesterolemia.

The administration of simvastatin (SVS) at 10 mg/kg, a cholesterol‐lowering drug also had demonstrated no significant effect on serum lipid profiles (TC, LDL, and TG) that mainly attributed by origin source, initial weight, feeding sessions, duration of the study, stress level and environmental conditions of experimental rats. The primary site of simvastatin action was at the liver to block the activity of HMGCoA reductase. However, the long‐term consumption of this drug may affect liver function, loss of appetite, myopathy with an elevation of creatinine kinase and rhabdomyolysis (Tiwari & Khokhar, 2014).

## 5 CONCLUSION

Effects of bioactive compounds such as monacolin K, naringin, tocopherol in this combination of selected functional food mixture (MSFF) may exert curative action on hypercholesterolemia via inhibition of lipid peroxidation, HMGCoA reductase and ACAT2 activities compared to the treatment using single ingredient. The beneficial effects of these functional ingredients should be advocated as an alternative treatment to combat hypercholesterolemia.

## 7 CONFLICT OF INTEREST

The authors declare no conflict of interest.

## 8 ETHICAL APPROVAL

All the procedures were performed according to the recommendation of the ethics committee by the Institutional Animal Care and Use Committee (IACUC) of Universiti Putra Malaysia (UPM) with AUP No: UPM/IACUC/AUPR084/2018 for the animal study protocol.

9

## Data Availability

The data that support the findings of this study are available from the corresponding author upon reasonable request.
